# Open access publishing: a girder in the success of the Scandinavian Journal of Trauma, Resuscitation and Emergency Medicine

**DOI:** 10.1186/1757-7241-19-7

**Published:** 2011-01-19

**Authors:** Hans Morten Lossius, Kjetil Søreide

**Affiliations:** 1 Department of Research, Norwegian Air Ambulance Foundation, Drøbak, Norway; 2 Department for Surgical Sciencies, Faculty of Medicine and Dentistry, University of Bergen, Bergen, Norway; 3 Department of Surgery, Stavanger University Hospital, Stavanger, Norway

## Editorial

The Scandinavian Journal of Trauma, Resuscitation and Emergency Medicine (SJTREM) has entered its third year as an open access international scientific on-line journal. The 2-year goal presented at the Editorial Board meeting in Copenhagen 2008 is achieved, with SJTREM now being indexed in PubMed, PubMed Central, Medline, Scopus and Google Scholar, and, presently accepted for tracking by Thompson Reuters (ISI). Submissions are steadily increasing, and the acceptance rate is per date approximately 40%. The average time from submission to first decision is 22 days, and more than 150 scientific papers have been published. One paper has been nominated for Faculty of 1000 post publication peer-review [1], the number of SJTREM papers cited in other journals are increasing, and all published papers reach a significant number of readers, far above what was achievable for the earlier paper version of SJTREM.

SJTREM converted into open access (OA) online publishing in July 2008 [2]. The decision was based on the importance of making research accessible for all, regardless of financial status or capabilities. This conversion resulted in a substantial rise in submissions, and not least citations. In line with the visions of saving more lives, the Norwegian Air Ambulance Foundation and the Laerdal Foundation for Acute Medicine have supported SJTREM by covering the article processing charges for the first and critical 2 years of establishing an independent scientific, open access journal of trauma, resuscitation and emergency medicine [3-5].

The OA conversion was timely. Scandinavian research founders have for the last two years been steadily moving from a supportive attitude for the OA principles, to making policy decisions that have a direct guidance to authors to publish OA. The Norwegian Research Council declared in 2009 that all public founded research should be published OA [6], and in Denmark the Open Access Committee has, on behalf of the Ministry of Science, Technology and Innovation, made clear recommendations for OA publishing [7]. But the most significant step till now was the decision of the Swedish Research Council and other major Swedish research founders to include an OA *mandate *for all its research grants from 2010 [8-10]. In line with this, universities in Scandinavia are moving towards OA publishing, and national libraries are following closely. Chalmers University of Technology was in 2010 the first Swedish university to take a strong Open Access mandate [11], and there is probably just a question of time before this is the common policy within most Scandinavian Universities.

The Scandinavian move is part of the wider global picture where mandates and funding mechanisms, constituting the equivalent of library budgets at many universities, are springing into life. With the support of European Commission, OA are evolving all over Europe http://www.openaire.eu/. Germany's Deutsche Forschungsgemeinschaft is encouraging all German universities to establish funding mechanisms and provides up to 75% of the costs of OA publishing [12]. As a result of such initiatives, the list of universities with central funds for OA publications is growing rapidly [13].

The signatories of the Compact for Open Access Publishing Equity commit "to the timely establishment of durable mechanisms for underwriting reasonable publication charges for articles written by its faculty and published in fee-based open-access journals and for which other institutions would not be expected to provide funds." The growing list of signatories include Harvard University, University of California at Berkley, Massachusetts Institute of Technology (MIT), University of Ottawa, Simon Fraser University, University de Barcelona, European Organization for Nuclear Research (CERN) and more http://www.oacompact.org/signatories/.

The costs of publishing in SJTREM are covered by article processing charges (APC). Till now, APCs in SJTREM have been kindly sponsored by The Norwegian Air Ambulance Foundation and The Laerdal Foundation for Acute Medicine. These sponsorships have been decisive for the rapid success of the Journal. From 1st February 2011, the APC payment will be agreed as part of the submission process, with the submitting author either agreeing to pay in full, requesting institutional membership or requesting a waiver. Payment will be requested once the manuscript has been editorially accepted for publication.

The number of BioMed Central Institutional membership in Scandinavia has reached 15 http://www.biomedcentral.com/inst/, and is growing. The BioMed Central's member institutions pay all or part (supporter members) of the APC for researchers affiliated at their institution.

The authors, Editorial Board (EB), and the invited peer-reviewers are the foundation for creating a high quality scientific journal. As Editor in Chiefs, we would like to express our gratitude and deep respect for all hours and scientific competence spent by all of you in establishing SJTREM as an meaningful source of knowledge for clinicians and scientist involved in trauma, resuscitation and emergency medicine world wide. The next years will be even more demanding, and a reinforced EB is ready to face these challenges. We hope that researchers will continue to consider SJTREM their choice to impart their achievements and opinions.

## Competing interests

The authors declare that they have no competing interests.
